# Organic Bee Pollen: Botanical Origin, Nutritional Value, Bioactive Compounds, Antioxidant Activity and Microbiological Quality

**DOI:** 10.3390/molecules17078359

**Published:** 2012-07-11

**Authors:** Xesús Feás, M. Pilar Vázquez-Tato, Leticia Estevinho, Julio A. Seijas, Antonio Iglesias

**Affiliations:** 1 Department of Organic Chemistry, Faculty of Science, University of Santiago de Compostela, E-27080, Lugo, Spain; Email: pilar.vazquez.tato@usc.es (M.P.V.-T.); julioa.seijas@usc.es (J.A.S.); 2 CIMO-Mountain Research Center, Agricultural College of Bragança, Polytechnic Institute of Bragança, Campus Santa Apolónia, E-5301-855, Bragança, Portugal; Email: leticia@ipb.pt; 3 Department of Anatomy and Animal Production, Faculty of Veterinary Medicine, University of Santiago de Compostela, E-27002, Lugo, Galicia, Spain; Email:antonio.iglesias@usc.es

**Keywords:** bee pollen, antioxidant capacity, bioactive compound, fatty acids, microbiological safety, organic food

## Abstract

Organic bee pollen (BP, n = 22) harvested from the Douro International Natural Park (DINP, Portugal) was studied. Nine botanical families were found in the mixture of the samples. The water activity and pH ranged 0.21–0.37 and 4.3–5.2, respectively. The BP analyses averaged 67.7% carbohydrates, 21.8% crude protein, 5.2% crude fat and 2.9% ash. The energy ranged from 396.4 to 411.1 kcal/100 g. The principal fatty acid found was linolenic, followed by linoleic acid, palmitic acid and oleic acid. The phenolic and flavonoid contents varied from 12.9 to 19.8 mg of gallic acid equivalents/g of extract and from 4.5 to 7.1 mg of catechin equivalents/g of extract, respectively. The scavenger activity and β-carotene bleaching assays values (EC_50_) were 3.0 ± 0.7 mg/mL and 4.6 mg/mL ± 0.9 mg/mL, respectively. *E. coli*, sulphite-reducing Clostridia, *Salmonella* and *S. aureus* were not found. Since there are studies indicating appreciable differences among BPs from different regions, the full characterization of BP from diverse origins still appears to be a sound research priority in order to obtain reliable data about this beehive product.

## 1. Introduction

Bee honey has been used by man since the beginning of Humanity. Although this is the most common beehive product, there are other products, such as bee pollen (BP), royal jelly, propolis and beeswax. These natural goods are well appreciated by consumers due to the high number of quality checks they go through, as well as for their dietetic and therapeutic qualities.

BP is the result of the agglutination of flower pollens; it is made by worker honey bees with nectar and salivary substances and stored at the hive entrance [1]. The collection of BP is a relatively recent development, dependent primarily on the basic concept of scraping pollen off of the bees’ legs as they enter the hive. When analyzing and studying the nutritional and therapeutic properties of BP, modern science has made it possible to specify its valuable antimicrobial [2], antifungal [3], antioxidant [4], anti-radiation [5], hepatoprotective [6], chemopreventive [7], anticancer [8] and antiinflammatory activities [9].

The major components of BP are carbohydrates, crude fibers, proteins and lipids at proportions ranging between 13 and 55%, 0.3 and 20%, 10 and 40%, 1 and 10%, respectively. Other minor components are minerals and trace elements, vitamins and carotenoids, phenolic compounds, flavonoids, sterols and terpenes [10]. In fact, BP is referred to as the “only perfectly complete food”, as it contains all the essential amino acids needed for the human organism. However, the composition of BP depends strongly on the plant source and geographic origin, together with other factors such as climatic conditions, soil type, and beekeeper activities.

Apiculture is valuable in social, environmental and economic terms and the conservation and preservation of this practice is essential. The quality and diversity of Portuguese’s landscape should be considered a valuable and supporting resource for apiculture to achieve international prominence and competitiveness. Nowadays, honey represents the most highly valued product, since Portugal further regulates the registration of honey (9 of 18), bearing the European Protected Designation of Origin (PDO) [11,12]. However, the collection of BP, which as a high quality product and used to be appreciated, currently suffers from marketing problems because there is a large sector of the public who are misinformed about its properties.

Presently, in the Portuguese continental territory there are 29 Special Protected Areas and 60 Sites of Community Importance classified according to the Council Directive 92/43/EEC which deals with the conservation of natural habitats and wild fauna and flora that are considered to be threatened in the European Union [13]. The management of such areas must be ecological, economical and socially sustainable; which makes apiculture one of the most promising activities to develop.

Organic apiculture is an ecologically based system, which encourages the use of good agricultural practices to maintain the balance and diversity of the agricultural ecosystem, and also promotes the sustainable use of natural resources, environmental quality, animal welfare and human health [14]. Organic beehive products are free from many problems, such as pollution fallout and chemical residues. Moreover, the use of the beehive products for therapeutic purposes demands it be harvested in areas with no organic contamination sources [15]. Today, concerns about traces of numerous toxic substances have prompted some demand for beehive products that are certified as organic [16]. However full characterization of BP is scarce and there is a lack of information about the characteristics of the product certified as organic [17]. 

The present study aims to characterize, for the first time, organic AP with respect to: (i) floral origin; (ii) physico-chemical (water activity and pH), nutritional (ash, protein, fat and carbohydrate) and energy value; (iii) fatty acid profile; (iv) bioactive compounds (phenolics and flavonoids); (v) antioxidant activity; and (vi) microbial safety (aerobic mesophiles, moulds and yeasts, fecal coliforms, *Escherichia coli*, sulphite-reducing *Clostridia*, *Salmonella* and *Staphylococcus aureus*). 

## 2. Results and Discussion

### 2.1. Palynological Identification

The BP profile analysis results allow us to determinate its floral origin. Table 1 shows the frequency of occurrence, range and mean values of the 11 pollen types identified in the 23 samples. The BP analyzed have between three (samples 3 and 4) and seven (sample 13) pollen types; the mean number is 4.8 with a SD of 1.0. Nine families of PL were found in the BP mixture of: *Cistaceae*, *Boraginaceae*, *Rosaceae*, *Fagaceae*, *Asteraceae*, *Fabaceae*, *Ericaceae*, *Mimosaceae* and *Myrtaceae.* None of the botanical families is represented in all the samples studied, since PL can vary according to the region where they are offered, a factor that depends on the available surrounding bee pasture in the apiary vegetation. 

**Table 1:** Frequency classes (presence, range and mean ± SD) of the pollen types in the organic apian pollen.

Family	Pollen type	Found (n ^a^)	Frequency (%)	Range (%)	Mean ± SD ^b^ (%)
*Cistaceae*	*Cistus*	17	77.3	5.2–90.6	44.0 ± 30.0
*Boraginaceae*	*Echium*	16	72.7	24.5–60.5	24.5 ± 18.7
*Rosaceae*	*Prunus*	12	54.5	0.8–10.3	5.8 ± 2.7
*Fagaceae*	*Castanea*	11	50.0	1.2–65.8	23.6 ± 22.6
*Asteraceae*	*Leontodon*	10	45.5	3.2–49.5	21.6 ± 12.3
*Fabaceae*	*Trifollium*	10	45.5	4.4–45.6	13.3 ± 12.3
*Ericaceae*	*Erica*	8	36.4	6.4–68.0	32.7 ± 24.4
*Fagaceae*	*Quercus*	7	31.8	1.2–16.0	8.3 ± 4.9
*Mimosaceae*	*Mimosa*	7	31.8	1.2–11.2	5.3 ± 3.6
*Myrtaceae*	*Eucalyptus*	4	18.2	1.3–5.6	3.2 ± 1.8
*Rosaceae*	*Rubus*	3	13.6	2.1–5.6	4.0 ± 1.8

^a^ sample size; ^b^ SD = standard deviation.

**Table 2:** Palynological spectrum of the total organic apian pollen.

Pollen Types		Samples																					
		1	2	3	4	5	6	7	8	9	10	11	12	13	14	15	16	17	18	19	20	21	22
*Rosaceae*	*Prunus*	5.0	ND	ND	ND	ND	9.8	0.8	5.4	2.8	5.9	7.1	ND	3.8	10.3	ND	6.0	ND	ND	6.7	ND	ND	6.7
		I					I	I	I	I	I	I		I	I		I			I			I
	*Rubus*	ND	ND	ND	ND	ND	ND	ND	ND	ND	ND	ND	ND	ND	ND	ND	3.1	ND	5.6	ND	4.4	ND	ND
																	I		I		I		
*Cistaceae*	*Cistus*	68.2	56.0	5.4	80.6	54.8	ND	90.6	16.8	69.8	6.5	ND	5.2	ND	11.5	32.8	13.2	65.0	ND	67.9	25.7	74.5	ND
		D	D	P	D	D		D	A	D	I		I		I	A	I	D		P	A	D	
*Boraginaceae*	*Echium*	2.5	23.2	26.6	18.2	ND	ND	5.4	19.8	2.6	18.7	48.9	ND	53.6	ND	49.6	17.1	21.2	ND	18.5	ND	6.9	60.5
		I	A	A	A			I	A	I	A	D		D		D	A	I		A		I	D
*Fagaceae*	*Castanea*	19.9	10.8	ND	12	18.8	65.8	ND	ND	24.8	ND	ND	ND	4.5	54.6	5.2	ND	ND	48.9	ND	ND	5.4	ND
		A	I		I	A	D			A				I	D	I			D			I	
	*Quercus*	ND	ND	ND	ND	1.2	ND	ND	ND	ND	9.4	7.8	16.0	5.3	ND	ND	ND	ND	ND	ND	12.5	ND	5.6
						I					I	I	A	I							I		I
*Fabaceae*	*Trifolium*	4.4	ND	ND	ND	ND	5.6	ND	ND	ND	ND	7.8	45.6	ND	ND	9.0	14.7	12.5	19.8	6.9	ND	6.8	ND
		I					I					I	D			I	I	I	A	I		I	
*Asteraceae*	*Leontondon*	ND	10.0	ND	ND	19.6	17.6	3.2	ND	ND	ND	28.4	25.4	17.8	23.6	ND	ND	ND	21.3	ND	49.5	ND	ND
			I			A	A	I				A	A	A	A				A		D		
*Ericaceae*	*Erica*	ND	ND	68.0	ND	ND	ND	ND	46.8	ND	54.3	ND	6.5	6.8	ND	ND	45.9	ND	ND	ND	ND	6.4	27.2
				D					D		D		I	I			D					I	A
*Myrtaceae*	*Eucalyptus*	ND	ND	ND	ND	5.6	ND	ND	ND	ND	ND	ND	ND	ND	ND	3.4	ND	1.3	ND	ND	2.5	ND	ND
						I										I		I			I		
*Mimosaceae*	*Mimosa*	ND	ND	ND	ND	ND	1.2	ND	11.2	ND	5.2	ND	1.3	8.2	ND	ND	ND	ND	4.4	ND	5.4	ND	ND
							I		I		I		I	I					I		I		

D: Dominant Pollen (>45%); A: Acessory Pollen (15%–45%), I: Isolated Pollen (<15%) and ND: not detected.

A full spectrum analysis of the total BP is given in Table 2. On the basis of palynological analysis, most of the samples were found to be heterofloral, due to their different colours and consequently different pollen types. However, in two samples the occurrence of over 80% of *Cistus* pollen type (samples 4 and 7) characterized them as unifloral. From the economical standpoint, the assessment of a monofloral origin may increase the commercial value of these BPs. In fact, it has been reported that bee pollen from *Cistus sp*. has anabolic and stimulatory effects on bone components in rats *in vitro* and *in vivo* [18,19,20], a potent anti-inflammatory activity [9], antiallergic action [21] and high antioxidative and scavenging abilities [22,23]. 

Bees forage different plants; thus, BP is always a mixture of different sources. However, in food control, pollen analysis is very efficient for the differentiation of BP produced in distinctly different geographical and climatic areas, as well as to ascertain the monofloral origin of BP obtained from intensively cultivated crops.

Moreover, palynology also allows scientists to infer the vegetation present in an area, and to date and ascertain any biodiversity changes, as for example the presence and distribution of invasive and/or exotic plants. Results showed that BP from the DINP contained *Mimosa* and *Eucalyptus* pollen types, found in 7 and 4 samples, respectively. 

### 2.2. Water Activity (a_w_), pH and Nutritional Composition

The a_w_ of BP samples is 0.31 (average) with a range of 0.21–0.37 and a SD of 0.04 (Table 3). The range obtained was typical of dehydrated foods and similar when compared to BP from Brazil (0.3–0.5; [24]) or from Spain (0.261–0.280; [25]). All the BP samples analysed were acidic, with a pH in the range of 4.3 and 5.2, with an average of 4.8 (±SD = 0.2). 

**Table 3:** Physico-chemical, nutritional and energetic values of organic bee pollen samples.

Sample	a_w_	pH	Ash	Proteins	Fat	Carbohydrates	Energy
S1	0.44	4.9	2.4	23.1	5.7	66.2	407.9
S2	0.45	4.6	3.6	21.4	5.0	67.5	400.5
S3	0.44	4.7	3.4	25.6	6.1	62.0	405.2
S4	0.35	5.2	2.6	23.0	4.5	67.2	401.3
S5	0.45	5.0	2.1	21.9	5.5	67.9	408.9
S6	0.54	5.1	2.1	25.6	4.7	64.7	402.8
S7	0.22	4.6	3.3	27.1	5.2	61.2	400.1
S8	0.21	4.3	2.0	23.0	5.0	67.3	406.3
S9	0.22	4.5	4.0	19.1	6.3	68.4	406.6
S10	0.41	5.1	2.0	21.0	4.9	69.6	406.7
S11	0.42	4.8	2.2	19.2	5.8	70.6	411.1
S12	0.41	4.8	3.0	21.1	4.3	69.2	399.9
S13	0.40	5.0	2.4	20.3	5.0	69.9	406.1
S14	0.40	4.5	2.9	19.5	4.8	70.5	403.3
S15	0.40	4.6	2.5	22.2	5.3	67.4	406.0
S16	0.32	4.9	2.8	20.6	4.8	69.5	403.2
S17	0.44	4.8	4.0	21.3	5.0	67.2	399.1
S18	0.51	4.5	4.0	19.7	4.3	69.7	396.4
S19	0.44	5.1	4.0	19.3	5.7	68.8	403.2
S20	0.32	4.8	2.4	23.7	6.3	64.8	411.0
S21	0.52	4.9	3.0	22.2	5.4	66.9	404.8
S22	0.37	4.5	3.2	19.4	5.0	70.2	403.1
Mean	0.39	4.8	2.9	21.8	5.2	67.6	404.3
^a^ SD	0.09	0.2	0.7	2.2	0.6	2.6	3.8
^b^ V_max_	0.54	5.2	4.0	27.1	6.3	70.6	411.1
^c^ V_min_	0.21	4.3	2.0	19.1	4.3	61.2	396.4

^a^ SD = standard deviation; ^b^ V_max_ = maximum value; and ^c^ V_min_ = minimum value.

Knowledge about the a_w_ in BP is useful to improve its conservation and storage by preventing the growth of molds and yeasts. If mold grows, it then ferments, resulting in a product with an off-taste, high levels of dead yeast, and ethanol that reduces the quality of this product. Furthermore, among the risks of consuming BP with high a_w_ and commonly stored at room temperature, one is contamination by fungi, which might produce carcinogenic mycotoxins [26].

The low pH and a_w_ inhibits the presence and growth of microorganisms and makes BP compatible with many food products. Both parameters are of great importance during the storage of BP as it influences its texture, stability and shelf life.

### 2.3. Nutrients Composition

The results of the basic nutrient composition and estimated energetic value (expressed on dry weight basis) obtained for the analyzed BP are shown in Table 3. The analysis of BP from DINP averaged 67.7% CH, 21.8% CP, 5.2% CF and 2.9% ash. The energy value of the analyzed BP ranged from 396.4 to 411.1 kcal/100 g (mean value ± SD = 404.3 ± 3.8 kcal/100 g). Those values confirm that BP is an excellent source of energy. 

The chemical composition and nutritional value of BP shows considerable variability between plant species. For example, pollens of pine, corn and bulrush contain 13.92; 36.59; and 31.93% total carbohydrates, 13.45; 20.32; and 18.90% proteins; 1.80; 3.7; and 1.16% lipids and 2.35; 4.90; and 3.80% total ash respectively [27]. Many factors are known to affect the nutrient content of BP, including climate, geography, apicultural practices and the genetic composition of the plant species. 

Generic BP composition data were considered sufficient for most purposes, but now the usefulness of BP-specific composition data is increasingly being acknowledged. We decided to use BP in the same form as it appears when commercialized by beekeepers, because it would be economically impossible for them to separate the pollens into families before selling it. It should be clear that when nutrient contents are significantly different among foods of the same species, those foods should be reported independently in food composition databases and other printed materials-including food labels-with their unique nutrient profiles [28].

**Table 4:** Fatty acid profile of the organic apian pollen.

Sample	C10:0	C16:0	C18:1n9c + t	C18:2n6c	C18:3n3	C20:0	C20:1c	Total ^a^	Other	∑SFA	∑MUFA	∑PUFA	∑TUFA	PUFA/SFA	ω6/ω3	ω3/ω6	TUFA/SFA	SFA/TUFA
S1	5.39	10.15	9.85	14.79	50.71	3.15	1.20	95.24	4.76	18.69	11.05	65.50	76.55	3.50	0.29	3.43	4.10	0.24
S2	7.68	7.83	9.42	17.02	48.19	0.51	3.10	93.74	6.26	16.02	12.52	65.21	77.72	4.07	0.35	2.83	4.85	0.21
S3	3.24	9.02	16.68	20.11	42.84	1.90	1.30	95.09	4.91	14.16	17.98	62.94	80.93	4.44	0.47	2.13	5.71	0.17
S4	3.87	10.45	20.61	18.66	40.08	ND	ND	93.66	6.34	14.32	20.61	58.74	79.34	4.10	0.47	2.15	5.54	0.18
S5	3.65	7.90	15.65	20.08	41.64	2.12	1.65	92.70	7.31	13.67	17.30	61.72	79.02	4.51	0.48	2.07	5.78	0.17
S6	3.87	10.45	20.61	18.66	40.08	ND	ND	93.66	6.34	14.32	20.61	58.74	79.34	4.10	0.47	2.15	5.54	0.18
S7	3.87	10.00	14.80	24.80	35.82	ND	2.15	91.43	8.57	13.87	16.95	60.61	77.57	4.37	0.69	1.44	5.59	0.18
S8	ND	30.05	4.63	5.94	55.73	ND	ND	96.35	3.65	30.05	4.63	61.67	66.29	2.05	0.11	9.39	2.21	0.45
S9	2.98	12.54	11.69	7.89	56.90	1.54	1.23	94.77	5.23	17.06	12.92	64.79	77.71	3.80	0.14	7.21	4.56	0.22
S10	ND	17.50	6.97	15.06	55.73	2.10	ND	97.36	2.64	19.60	6.97	70.79	77.76	3.61	0.27	3.70	3.97	0.25
S11	3.55	10.56	13.34	19.08	42.69	1.12	2.01	92.35	7.66	15.23	15.35	61.77	77.12	4.06	0.45	2.24	5.06	0.20
S12	7.68	8.12	11.40	17.80	45.70	0.51	ND	91.21	8.79	16.31	11.40	63.50	74.90	3.89	0.39	2.57	4.59	0.22
S13	8.27	20.31	11.22	23.26	30.25	0.10	ND	93.40	6.60	28.68	11.22	53.51	64.73	1.87	0.77	1.30	2.26	0.44
S14	6.87	17.15	4.80	24.80	30.24	0.23	0.33	84.42	15.58	24.25	5.13	55.04	60.17	2.27	0.82	1.22	2.48	0.40
S15	3.25	10.56	17.88	22.11	37.53	ND	3.35	94.66	5.34	13.81	21.22	59.63	80.86	4.32	0.59	1.70	5.86	0.17
S16	6.87	22.15	4.80	24.80	25.82	ND	ND	84.43	15.57	29.02	4.80	50.61	55.42	1.74	0.96	1.04	1.91	0.52
S17	3.33	16.45	9.56	17.76	44.65	0.45	0.22	92.42	7.58	20.23	9.78	62.41	72.19	3.08	0.40	2.51	3.57	0.28
S18	5.24	20.64	7.89	24.46	34.15	ND	ND	92.37	7.63	25.88	7.89	58.60	66.49	2.26	0.72	1.40	2.57	0.39
S19	4.24	10.62	11.98	21.11	42.33	2.12	2.31	94.71	5.29	16.98	14.30	63.44	77.73	3.74	0.50	2.01	4.58	0.22
S20	8.47	20.15	5.80	20.80	28.83	1.04	1.33	86.42	13.58	29.66	7.13	49.63	56.76	1.67	0.72	1.39	1.91	0.52
S21	4.57	8.95	18.91	17.79	43.11	ND	ND	93.31	6.69	13.51	18.91	60.90	79.80	4.51	0.41	2.42	5.91	0.17
S22	6.00	10.54	12.79	20.36	40.53	ND	0.34	90.56	9.44	16.54	13.13	60.89	74.02	3.68	0.50	1.99	4.48	0.22
Mean	4.68	13.73	11.88	18.96	41.52	0.77	0.93	92.47	7.53	19.18	12.81	60.48	73.29	3.44	0.50	2.65	4.23	0.27
* SD	2.33	5.93	5.04	4.91	8.65	0.96	1.09	3.43	3.43	5.89	5.34	4.92	7.94	0.99	0.21	1.98	1.41	0.12
** V_max_	8.47	30.05	20.61	24.80	56.90	3.15	3.35	97.36	15.58	30.05	21.22	70.79	80.93	4.51	0.96	9.39	5.91	0.52
*** V_min_	ND	7.83	4.63	5.94	25.82	ND	ND	84.42	2.64	13.51	4.63	49.63	55.42	1.67	0.11	1.04	1.91	0.17

^a^ (Calculated value). * SD = standard deviation. ** V_max_ = maximum value. *** V_min_ = minimum value and ND = no detected. Capric acid (C10:0); Palmitic acid (C16:0); Oleic acid (C18:1n9c + t); Linoleic acid (C18:2n6c); α-Linolenic acid (C18:3n3); Arachidic acid (C20:0); Eicosenoic acid (C20:1c). SFA: Saturated fatty acids (C10:0 + C16:0 + C20:0); MUFA: Monounsaturated fatty acids (C18:1n9c + t + C20:1c); PUFA: Polyunsaturated fatty acids (C18:2*n*6 + C18:3*n*3); TUFA: Total unsaturated fatty acids (∑MUFA + ∑PUFA).

### 2.4. Fatty Acid Profile Determination

The percentage of fatty acid (FA) composition (%) of BP samples from DINP are shown in Table 4. Seven FAs were identified and quantified in the pollen samples: capric acid (CPA, C10:0); palmitic acid (PA, C16:0); oleic acid (OA, C18:1n9c + t) linoleic acid (LA, C18:2n6c); linolenic acid (LNA, C18:3n3); arachidic acid (AC, C20:0) and eicosenoic acid (EA, C20:1c). The principal fatty acid in the BP samples was LNA, ranging between 25.82% and 56.90%. It was followed by LA (5.94% to 24.80%), PA (7.83 to 30.05) and OA (4.63 to 20.61). CA, EA and AC were not found in all AP samples and averaged 4.68%, 0.93% and 0.77%, respectively. In the literature, there are a number of studies indicating appreciable differences among FA compositions of BPs from different regions or countries. Therefore, the full characterization of BPs of diverse origins still appears to be a sound research priority to obtain a reliable data on this valuable beehive product. 

The total saturated FA (SFA = C10:0 + C16:0 + C20:0), monounsaturated FA (MUFA = C18:1n9c + t + C20:1c), polyunsaturated FA (PUFA = C18:2*n*6 + C18:3*n*3), total unsaturated FA (TUFA = ∑MUFA + ∑PUFA) and the PUFA/SFA, SFA/TUFA ratios, and ω−6/ω−3, ω−3/ω−6, obtained for the AP samples are shown in Table 4. The analysed samples present a level of TUFA between 55.42% and 88.93% of the total (calculated value) FA. In all cases, PUFA is significantly higher than MUFA and SFA. The profiles obtained for the analyzed organic BP from the Portuguese DINP were similar to the data from a report by Shawer *et al*. [29], on Spanish pollens but are slightly different from those of Poland, Korea and China [30]. The LA and LNA are key compounds for cell membranes and are associated with brain function and neurotransmission. These FA also play an important role in the transference of the O_2_ to blood plasma, in the synthesis of hemoglobin and in cellular division [31,32]. Moreover, FA from ω−6 series are biogenetic precursors of some physiologically important thromboxanes, leukotrienes and prostaglandins hormones, which are related to the inflammatory response. The nutritional value of essential ω−3 and ω−6 FA is also widely known for its health benefits [33].

### 2.5. Bioactive Compounds and Antioxidant Activity

**Figure 1:**
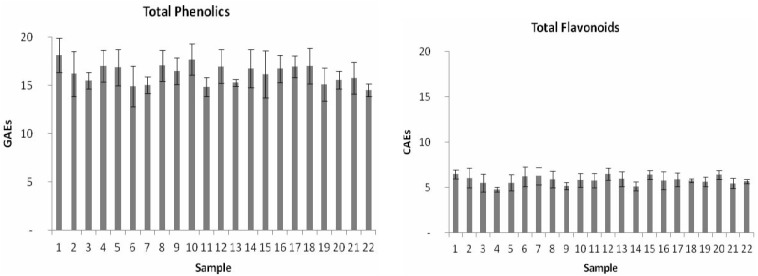
Total phenolics [TPC; in mg of gallic acid equivalents per g of organic apian pollen extract (GAEs)] and total flavonoids [TFC; in mg of catechin equivalents per g of organic bee pollen extract (CAEs)] of the analyzed samples.

The BPs were screened for their bioactive compounds, namely phenolics and flavonoids, and results are presented in Figure 1. Concentrations of TPC and TFC varied from 12.9 to 19.8 (mean value ± SD = 16.4 ± 2.0 GAEs) and from 4.5 to 7.1 (mean value ± SD = 5.8 ± 0.8 CAEs) respectively. Results showed that the concentration of TFC shows a minor variation, if we compare it to the results obtained for TPC.

**Figure 2:**
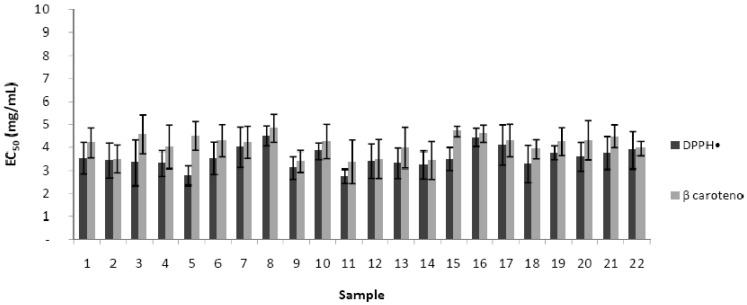
EC_50_ values (mg/mL) obtained for the antioxidant activity by scavenging of 2,2-diphenyl-1-picryl-hydrazyl radicals (DPPH) and β-carotene bleaching assay (BCB) in the extracts from organic apian pollen samples.

Previous works verified that antioxidant activity is related to the amount of flavonoids and phenolic compounds [34]. In this research, the correlations found were: TPC-DPPH (R = 0.60), TPC-BCB (R = 0.29), TFC-DPPH (R = 0.54) and TFC-BCB (R = 0.58). Figure 2 summarises the results obtained for antioxidant activity of the MeOH-APE. The use of at least two methods is recommended to assess and compare the antioxidant capacity of a sample [35]. In the present work antioxidant properties of the MeOH-APE were evaluated by the DPPH method and BCB assay. The scavenger activity of the free DPPH• was expressed in terms of EC_50_, that is the amount of antioxidant necessary to decrease by 50% the initial DPPH concentration. Lower values indicate better antioxidant capacity of the MeOH-APE. The EC_50_ values ranged from 2.0 mg/mL to 4.3 mg/mL, with a mean value of 3.0 ± 0.7 mg/mL. The results obtained (EC_50_) in BCB assay averaged 4.6 mg/mL with a range of 3.1 to 5.9 mg/mL and a SD of 0.9. 

As expected the β-carotene bleaching protection of \ the samples was lower than that provided by the TBHQ standard (79.3% at 2 mg/mL). In the DPHH assay, the results are expressed as the ratio percentage of the absorbance decrease of DPPH· solution in the presence of MeOH-APE, and the free radical-scavenging activity of the extracts is attributed to their hydrogen-donating ability [36]. In the BCB assay, the LA free radical, formed upon the abstraction of a hydrogen atom from one of its diallylic methylene groups, attacks the highly unsaturated *β*-carotene and undergoes rapid discolouration in the absence of an antioxidant. The presence of antioxidants in the MeOH-APE can hinder the extent of BCB by neutralizing the linoleate-free radical and other free radicals formed in the system [37]. As the molecules lose their double bonds by oxidation, the compound loses its characteristic orange colour, a fact that can be monitored spectrophotometrically.

The experimental results of antioxidant activity of MeOH-APE from the BP from DINP were similar to the data by Campos *et al*. [34] from pollen harvested in Portugal and New Zeland (EC_50_ of 40 to 500 μg/mL) but superior to those found by Leblanc *et al*. [38] in Sonoran Desert pollen, (antioxidant rates from 19% to 90%). 

Comparing the results obtained from BP in the present work with values previously observed in honey, it is possible to conclude that the antioxidant activity of MeOH-APE is higher than honey (EC_50_ of 37.03, 39.25 and 75.51 mg/mL for dark, amber and light honeys, respectively) [39]. 

### 2.6. Microbial Quality

BP samples were subjected to study in what concerns their aerobic mesophiles, moulds and yeasts, fecal coliforms, *E. coli*, clostridia spores, *Salmonella* and *S. aureus* on different selective microbiological media. Based on the obtained results (Table 5) it could be concluded that organic BP samples from DINP represent a product of high microbiological quality. This could be attributed to the nature of such foodstuffs in relation to their origin, processing and handling. Examined samples did not show any presence of *E. coli*, sulphite-reducing Clostridia, *Salmonella* and *S. aureus.* Fecal coliforms were only detected in two samples. In the work of Serra and Escola [40], honeybee-collected pollens were contaminated with high number of moulds, coliforms and Lancefield group D *Streptococci*.

**Table 5:** Microbial analyses of the organic apian pollen.

Sample	Aerobic mesophiles	Moulds and yeasts	Fecal coliforms	*E. coli* (in 1 g)	Sulphite-reducing clostridia (in 0.01 g)	*Salmonella* (in 25 g)	*S. aureus* (in 1 g)
S1	900	320	<1	absent	absent	absent	absent
S2	560	300	<1	absent	absent	absent	absent
S3	832	220	<1	absent	absent	absent	absent
S4	1500	120	<1	absent	absent	absent	absent
S5	500	240	<1	absent	absent	absent	absent
S6	2450	132	<1	absent	absent	absent	absent
S7	1700	2800	20	absent	absent	absent	absent
S8	650	3560	30	absent	absent	absent	absent
S9	1000	1672	<1	absent	absent	absent	absent
S10	<10	<10	<1	absent	absent	absent	absent
S11	<10	450	<1	absent	absent	absent	absent
S12	1980	1230	<1	absent	absent	absent	absent
S13	750	1500	<1	absent	absent	absent	absent
S14	2109	420	<1	absent	absent	absent	absent
S15	<10	100	<1	absent	absent	absent	absent
S16	450	220	<1	absent	absent	absent	absent
S17	670	<10	<1	absent	absent	absent	absent
S18	1250	2500	<1	absent	absent	absent	absent
S19	2450	3000	<1	absent	absent	absent	absent
S20	790	900	<1	absent	absent	absent	absent
S21	<10	100	<1	absent	absent	absent	absent
S22	<10	<10	<1	absent	absent	absent	absent

Taking into account the nutrient content, a variety of microorganisms could grow in BP. If harvest, storage and marketing practices are not appropriate, microorganisms might develop in it as happens in dehydrated foods. The quantification of quality parameters for commercial purposes (aerobic mesophiles and moulds and yeasts) is generally lower than that reported by other authors. Hervatin [41] found mould and yeast levels greater (10^4^ CFU/g) than those obtained in the present study. From the microbiological point of view, the low values of moulds and yeasts are most probably related to environmental conditions, and are indicative of an appropriate management of organic apiaries.

Microbiological criteria provide guidance on the acceptability of foodstuffs and their manufacturing processes. From the hygienic point of view, microbiological safety is the main quality criterion in BP. Destruction of bacteria by irradiation, ozone treatments or chemical fumigants is not necessary and can lead to toxic residues [10]. The microbiological content should correspond to the hygienic standards: *Salmonella* (absent/10 g), *S. aureus* (absent/1 g), *Enterobaceteriaceae* (Max. 100/g), *E. coli* (absent/1 g), total aerobic plate count (<100,000/g) and moulds and yeast (<50,000/g) [1]. 

Most primary producers are not directly affected, as specific microbiological criteria have not been set for beehive products. However, beekeepers may be affected indirectly if their customers require changes to specifications, as a result of improvements in production hygiene and selection of raw materials for health purposes.

## 3. Experimental

### 3.1. Chemicals

2,2-Diphenyl-1-picrylhydrazyl (DPPH) was obtained from Alfa Aesar (Ward Hill, MA, USA). 3,4,5-Trihydroxybenzoic acid (gallic acid; GA), *tert*-butylhydroquinone (TBHQ), butylated hydroxyanisole (BHA), polyoxyethylene (20) sorbitan monooleate (Tween 80), α-tocopherol, β-carotene, petroleum ether and ethanol were obtained from Sigma Chemical Co. (St. Louis, MO, USA). Sodium sulfate (Na_2_SO_4_, anhydrous, powder, extra pure), H_2_SO_4_, KOH, aluminium chloride (AlCl_3_)_,_ NaNO_2_ and NaOH were purchased from Acros Organic (Geel, Belgium). Capric acid (CPA, C10:0) [CAS 334-48-5]; palmitic acid (PA, C16:0) [CAS 57-10-3]; oleic acid (OA, C18:1n9c + t) [CAS 112-80-1 CAS 112-79-8]; linoleic acid (LA, C18:2n6c) [CAS 60-33-3]; linolenic acid (LNA, C18:3n3) [CAS 463-40-1]; arachidic acid (AC, C20:0) [CAS 506-30-9] and eicosenoic acid (EA, C20:1c) [CAS 5561-99-9]) were purchased from Sigma-Aldrich (Tres Cantos, Spain). The Folin-Ciocalteu reagent (FCR), chloroform (CHCl_3_) and sodium carbonate (Na_2_CO_3_), were obtained from Merck (Darmstadt, Germany). Methanol (MeOH) and hexane were obtained from Pronolab (Lisboa, Portugal). High purity water (18 MΩ cm), which was used in all experiments, was obtained from a Milli-Q purification system (Millipore, Bedford, MA, USA).

### 3.2. Apian Pollen Material

Twenty-two (n = 22) typical bee pollen samples (AP), from *Apis mellifera*, were collected by beekeepers from different apiaries, located inside the Portuguese territory declared as Douro International Natural Park (IDNP). They were obtained using bottom-fitted pollen traps in May of 2010. A 10-day-cycle of pollen trapping installation was employed in order to avoid making pollen trapping difficult to bees: pollen was trapped for 5 days and after that period it was stopped for the next 5 days [42].

After the beekeepers dried the harvested material, a single 200 g jar of bee pollen was delivered to the Microbiology Lab, where it was stored in a dark place at room temperature (±20 °C) until analysis, which occurred no longer than one month after the extraction from the hives by beekeepers. All BP samples showed no sign of fermentation or spoilage.

### 3.3. Sample Floral-Type Identification

The determination of the frequency of pollen load (PL) classes, were determined in the BP, in order to ascertain the floral origin and to obtain a complete pollen spectrum. The botanical origin of the BP was based on the method proposed by Almeida-Muradian *et al*. [43]. Briefly, the analyses are based on the separation according to colour of PL from 2 g of BP. The PL counted were from 287 to 387 (mean value ± standard deviation = 335 ± 29 %). Each subsample was weighed to calculate its percentage in the main BP. Three slides of each subsample were prepared by washing the PL in 50% ethanol and using glycerin and paraffin for permanent preparations. The examination of the slides was carried out with a Leitz Diaplan microscope (Leitz Messtechnik GmbH, Wetzlar, Germany) at ×400 and ×1,000. In order to recognise the pollen grains, we used the reference collection of the CIMO-Mountain Research Center (Agricultural College of Bragança) and different pollen morphology guides.

### 3.4. Water Activity and pH

The water activity (a_w_) was measured using a model Rotronic Hygroskop DP. For pH, 5 g of grounded AP were diluted with 20 mL of distilled water and mixed thoroughly. The pH values for these samples were measured using a digital pH Meter (pH 526 Multical, WTW, Weilheim, Germany).

### 3.5. Chemical Composition and Nutritional Value

Chemical composition of the BP were analysed according to the AOAC procedures [44]. The ash content was determined after incineration at 600 ± 15 °C, in a SNOL 8.2/1100-1 electric laboratory furnace (AB “Umega”, Utena, Lithuania). Nitrogen content (N) was determined using the Kjeldahl method (230-Hjeltec Analyzer, Foss Tecator, Höganäs, Sweden). The crude protein (CP) content was calculated using the conversion factor of 5.6 (N × 5.6). The crude fat (CF) was determined by gravimetry after extraction with petroleum ether using an automatic Soxtec device (FOSS, Soxtec^TM^ 2050, Höganäs, Sweden). The total carbohydrate (CH) contents were obtained by difference. Ash, CP, CF and CH contents of AP were expressed as a percentage of the original sample on a dry weight basis (g/100 g sample). The total energy (kcal/100 g) was estimated using the Atwater coefficients (4 kcal/g for CP and CH, 9 kcal/g for CF) [45]. 

### 3.6. Fatty Acid Profile Determination

Fatty acid methyl esters (FAMEs) were prepared from the extracted CF fraction by transesterification using MeOH in the presence of H_2_SO_4_ as follows: A sample containing 20 ± 50 mg of lipids was redissolved in 0.75 mL *n*-hexane; then 0.1 mL of 2 N KOH in MeOH was added and the solution was mixed for 2 min in a vortex mixer (Model Reax 2000, Schwabach, Germany), dried over anhydrous Na_2_SO_4_ and left for 25 min. After phase separation the upper layer of *n*-hexane containing the FAMEs was removed and immediately injected into the gas-chromatograph (GC). Quantitative and qualitative analysis of FAMEs, was performed on a DANI model GC 1000 coupled flame-ionization detector (FID) equipped with a Macherey-Nagel (OPTIMA 225: 50% cyanopropyl-methyl–50% phenylmethylpolysiloxane) column (30 m × 0.32 mm ID × 0.25 μm *df*). The carrier gas was H_2_ at a pressure of 0.61 bar, and the split ratio was 1:40. The flow rate of the carrier gas was set at 4.0 mL/min. A thermal gradient from 170 to 240 °C at 3.5 °C/min was used with the injector and FID temperatures at 240 °C. The injection volume was 1 µL. FAMEs were identified by comparing the retention times of the peaks of the sample with those of known reference esters. Fatty acid (FA) composition was expressed as percent of major FAMEs from the peak areas with an integrator. Results were recorded and processed using CSW 1.7 software (DataApex 1.7).

### 3.7. Bioactive Compounds Quantification

#### 3.7.1. Extracts Preparation

Extracts were prepared according to procedures from Morais *et al*. [4] with modifications. Briefly, BPs were ground and a portion of the obtained powder was mixed (1:2) (w/v) with MeOH, sonicated for 30 min. and left to macerate for 48 h at room temperature. After this time, the solution was centrifuged for 5 min at 13,000 g (4 °C). The centrifugal supernatant was evaporated under reduced pressure by a rotavapor system consisting of a rotary vacuum evaporator (Heidolph VV. 2000, Leuven, Belgium) equipped with a water bath and a B169 vacuum pump (Büchi, Flawil, Switzerland). The residue was dissolved in H_2_O and lyophilised. Finally, the obtained AP extract (APE) was stored at −80 °C, for further analysis.

#### 3.7.2. Total Phenolic Content (TPC)

The total phenolic content in the BPE were recorded by FCR as described by Moreira *et al*. [46]. Briefly, a dilute solution of each BPE in MeOH (MeOH-APE; 500 μL of 1:10 g/mL) was mixed with 500 μL of the FCR and 500 μL of Na_2_CO_3_ (10% w/v). After incubation in dark at room temperature for 1 h, the absorbance of the reaction mixture at 700 nm is determined against the blank (the same mixture without the MeOH-APE) using a Unicam Helios Alpha UV-visible spectrometer (Thermo Spectronic, Cambridge, UK). GA standard solutions (0.01–0.08 mM) were used for constructing the calibration curve (y = 2.3727x + 0.0022; R^2^ = 0.9998). Total phenols content were expressed as mg of GA equivalents per g of BPE (GAEs).

#### 3.7.3. Total Flavonoid Content (TFC)

For flavonoid contents the aluminium chloride method was used. Briefly, MeOH-BPE (250 μL) was mixed with 1.25 mL of distilled H_2_O and 75 μL of a 5% NaNO_2_ solution. After 5 min, 150 μL of a 10% AlCl_3_·H_2_O solution was added. After 6 min, 500 μL of 1 M NaOH and 275 μL of distilled H_2_O were added to the mixture and vortexed. The intensity of the pink colour of the reaction mixture at 510 nm is determined against the blank (the same mixture without the MeOH-BPE). Catechin (CA) standard solutions (0.022–0.34 mM) were used for constructing the calibration curve (y = 0.9689x − 0.0092; R^2^ = 0.9987). The total flavonoids content (TFC) was expressed as mg of CA equivalents per g of BPE (CAEs).

### 3.8. Antioxidant Activity

#### 3.8.1. Scavenging of DPPH Radicals

The scavenging of DPPH· was assayed following the method described by Ferreira *et al*. [39]. Various concentrations of MeOH-APE (300 μL) were mixed with 2.7 mL of MeOH solution containing DPPH• (6 × 10^−5^ mol/L). The mixture was shaken vigorously and left to stand for 60 min in the dark (until stable absorption values were obtained). The reduction of the DPPH· was measured by continuously monitoring the decrease of absorption at 517 nm. The radical-scavenging activity (RSA) was calculated as a percentage of DPPH discoloration using the equation: %RSA = [(A_DPPH_ − A_S_)/A_DPPH_] × 100, where A_S_ is the absorbance of the solution when the MeOH-BPE has been added at a particular level and A_DPPH_ is the absorbance of the DPPH solution. The MeOH-BPE concentration providing 50% of radical scavenging activity (EC_50_) was calculated by interpolation from the graph of RSA percentage against extract concentration. The standards used were BHA and α-tocopherol. 

#### 3.8.2. β-Carotene Bleaching (BCB) Assay

The antioxidant activity of the MeOH-BPE was evaluated by the BCB assay, as described by Ferreira *et al*. [39]. A solution was prepared by dissolving 2 mg of β-carotene in 10 mL of CHCl_3_. Afterwards 2 mL of the aforesaid solution was pipetted into a 100 mL round-bottom flask. Then the organic solvent was removed at 40 °C under vacuum. 40 mg of LA, 400 mg of Tween 80 emulsifier and 100 mL of distilled H_2_O were added to the flask. The mixture was shaken and 4.8 mL of this emulsion were transferred into different test tubes containing 200 μL of different concentrations of the MeOH-BPE. The tubes were shaken and incubated at 50 °C in a water bath. As soon as the emulsion was added to each tube, the zero time absorbance was measured at 470 nm. Absorbance readings were then recorded at 20-min intervals until the control sample had changed color. A blank, devoid of β-carotene, was prepared for background subtraction. Lipid peroxidation inhibition (LPO) was calculated using the following equation: LPO = (β-carotene content after 2 h of assay/initial β-carotene content) × 100. The MeOH-BPE concentration providing 50% antioxidant activity (EC_50_) was calculated by interpolation from the graph of antioxidant activity percentage. TBHQ was used as standard. 

### 3.9. Microbiological Determinations

Microbiological determinations were carried out as described previously [47]. In short, 10 g of each BP were aseptically taken and homogenized using a pre-sterilized Stomacher Lab-Blender (Seward type 400, London, UK) for 3 min with 90 mL of pre-chilled (4 ± 0.5 °C) sterile peptone-physiological saline solution [0.1% neutral peptone + 0.85% NaCl (Merck, Darmstadt, Germany) in sterile deionized H2O, pH = 7.0 ± 0.05]. Decimal serial dilutions were prepared from this homogenate in the same chilled sterile diluents (1:10, by vol). The aerobic mesophile were determined using plate count agar (PCA), by counting the colony forming units (cfu/g of BP) after incubating the plates at 30 °C for 48 h. Moulds and yeasts counts followed the protocol of International Organization for Standardization (ISO) [48]. For sulphite-reducing clostridia counting, aliquots of 10, 5, 1 and 0.1 mL of the initial suspension were added to an empty tube, thermally treated at 80 °C for 5 min and covered with SPS (sulphite-polymixin-sulfadiazine) agar media, tubes were incubated at 37 °C for 5 days. Fecal coliforms were enumerated by the Most Probable Number (MPN) technique defined in the protocol ISO [49]. The positive results for fecal coliforms were studied for *E. coli*. Enumeration was made on Eosin Methylene Blue Agar-EMB Agar (Oxoid Inc., London, UK) incubated at 35 °C for 24 h. *Salmonella* detection followed the ISO protocol [50]. *Staphylococcus aureus* detection followed the protocol of [51]. All microbial tests were performed in triplicate.

## 4. Conclusions

Under European regulations [52], any claims of health or nutritional benefits of a food product must be supported by science. Different health claims can be made for BP: (a) long term ingestion of pollen and special pollen preparations can improve the physical performance and fitness of sportsmen and elderly people and (b) pollen intake can improve gut, gastroenterological and liver health. BP composition data were considered sufficient for most purposes, but now the usefulness of BP-specific composition data is increasingly being acknowledged, since the composition of BP varies greatly as a result of collection from different geographic regions, the time of collection, and the various species of vegetation from which the pollen is harvested by honeybees. The results obtained in this study demonstrated that BP constitutes a good source of healthy compounds, namely, phenolics, and suggests that it might be useful in prevention of diseases in which free radicals are implicated. Portuguese organic BP from DINP is nutritionally well-balanced and revealed high levels of healthy fatty acids and good PUFA/SFA ratios. Microbiologically, the commercial quality was considered good and all samples showed negative results for toxigenic species. The consumption of BP can be beneficial for the health and, as such, the investigation of its chemical, nutritional and microbiological composition will contribute to the assessment of this natural product. BP is a food supplement with potentially beneficial effects for humans.
